# Spatio-temporal partitioning and coexistence between leopard (*Panthera pardus fusca*) and Asiatic lion (*Panthera leo persica*) in Gir protected area, Gujarat, India

**DOI:** 10.1371/journal.pone.0229045

**Published:** 2020-03-11

**Authors:** Rohit Chaudhary, Nazneen Zehra, Azra Musavi, Jamal Ahmad Khan

**Affiliations:** 1 Department of Wildlife Sciences, Aligarh Muslim University, Aligarh, India; 2 Centre for Woman’s Studies, Aligarh Muslim University, Aligarh, India; Sichuan University, CHINA

## Abstract

Time and space are essential niche dimensions along which species tend to coexist. We assessed spatiotemporal resource partitioning between leopards and lions and hypothesized the differential use of spatiotemporal resources by leopards with respect to lions. We used a systematic camera trap survey to collect the data at 50 sites. The data were analyzed using overlap indices, and non-parametric test statistics to assess the spatiotemporal associations. Leopard and lion were crepuscular and nocturnal in their activity pattern. They did not segregate temporally and showed substantially high overlap and strong temporal association. Leopard segregates with lion spatially by overlapping less and showing no association in space use at specific camera trap sites. Leopards showed preference for dense habitats, while the lion preferred both dense and open habitats. Leopard showed moderate-overlap and positive association with key prey species, i.e., chital and sambar. Lion, however showed low site-specific overlap and negative association with its crucial prey species, i.e., sambar and wild pig. We conclude that site-specific spatial partitioning along with differential affinities for habitat is helping leopards to partition their spatio-temporal resources with lions and hence facilitate coexistence of leopards with lions in Gir forest.

## Introduction

Interspecific competition is among the major forces responsible for shaping the community structure and functioning [1]. Interspecific competition could be interference, where the dominant member in the guild can directly affect a subordinate member of the guild or exploitative where a dominant member of the guild may reduce resource availability without interacting directly with a subordinate member of the guild [2]. Gauss's exclusion principle states that species with similar resource requirements cannot coexist, and one of the sympatric species will be excluded [3,4]. Coexistence between sympatric species is possible when they partition themselves along the spatial, temporal, or dietary axis to reduce the ecological overlap [5,6]. Therefore, resource partitioning is among the key processes responsible for the coexistence of sympatric species.

Large carnivores owing to their predatory behavior and being at the top position can affect community structure disproportionately [7]. Due to their morphological similarities and similar resource requirements, sympatric large carnivores often result in higher overlap in resource use and could end up in competition. Competitive interactions among large carnivores are mostly asymmetrical when a dominant member of the guild can affect the abundance, distribution, and resource use patterns of the subordinate members of the guild [8]. Due to the substantial habitat loss, much of the carnivore population now exists in fragmented habitats, where the interspecific interactions may be intense [9].

Therefore, current competition in large carnivore guild cannot be ignored, and hence resource partitioning studies play an essential role in understanding how subordinate members of the guild partition their resources and coexist. Resource partitioning among large carnivores can be achieved through a variety of ecological processes such as dietary partitioning [10] and spatiotemporal partitioning [9,11]. Recent studies have also illustrated the importance of adjustment in fine space use vis-à-vis time between members of the carnivore guild to facilitate the resource partitioning. Such fine adjustment includes avoidance of risky habitat type where the encounter with a dominant competitor is more [12], avoiding hourly activity of dominant member [13] and avoidance of area with high temporal activity [14].

Leopard (*Panthera pardus fusca*) and Asiatic lion (*Panthera leo persica*) are two large carnivores that occur sympatrically in Gir Wildlife Sanctuary and National Park (Hereafter Gir). This guild provides a unique opportunity to assess resource partitioning due to the existence of the sole population of Asiatic lions in Gir. There have been studies on resource partitioning and coexistence among lions and leopards from the African ecosystem [15–24], but no study has been conducted yet regarding how leopard and Asiatic lion segregate resources and coexist. Zehra et al. [25] found that leopard and lion overlap very high (>90%) in their diet, which indicate the limited role of the diet in defining resource partitioning between these two predators. However, space use and activity pattern may possibly play an important role in resource partitioning between these two predators. Further, Gir harbours high densities of both leopards (19.8 individual/ 100 km^2^) [26] and lions (15 individuals / 100 km^2^) [27]. At such high densities, there is a high probability of direct encounters between leopards and lions. Spatiotemporal partitioning is among one of the key processes for predator-predator coexistence by minimizing the direct encounters [28]. Hence understanding how leopard and lion partition their space and time is important to understand their coexistence.

The goal of the present study was to understand spatiotemporal resource partitioning between the leopard and Asiatic lion. We asked two research questions in this study i) How do leopards partition resources spatiotemporally with lions and coexist? ii) Among space and time axis, which one contributes more to the coexistence of leopard with the lion? Due to smaller body size, leopards are subordinate to lions in Gir, and therefore we hypothesize that leopards will use space and time differently than lions in Gir. Prediction for this hypothesis was that leopard would show less spatial and temporal overlap, differential habitat preference than lions, and also show no association in space use and activity pattern with lions. Further, Lovari et al. [29] have quantified that tigers and leopards do not segregate along single niche dimensions, and partitioning along the three major niche dimensions (time, space, and food) contributes to their coexistence. Therefore, our second hypothesis was that both space and time will contribute to the coexistence of leopard and lion. The major objectives of the study were 1) To assess the pattern of space and time use by leopard and lion 2) To assess overlap and partition of space and time by leopards and lions.

## Materials and methods

Present was a field study conducted in a Gir protected area which includes Gir National Park and Wildlife Sanctuary. Permission for field work was granted by Principle Chief Conservator of Forest and Chief Wildlife Warden, Gujarat, India which is competent authority to grant permission in protected area. Permit number for the study was WLP/28/C/594-95/2016-2017. No specific permit was required for particular locations or activity. One of the studied species i.e., Asiatic lion comes under endangered category but present study did not include any direct handling since data were collected through camera trapping which is a non-invasive method.

### Study area

The present study was carried out in Gir Wildlife Sanctuary and National Park, situated in the western part of India. Forest type in Gir comes under very dry teak forest [30] [For more details, see 31].

### Data collection

**Fig 1:**
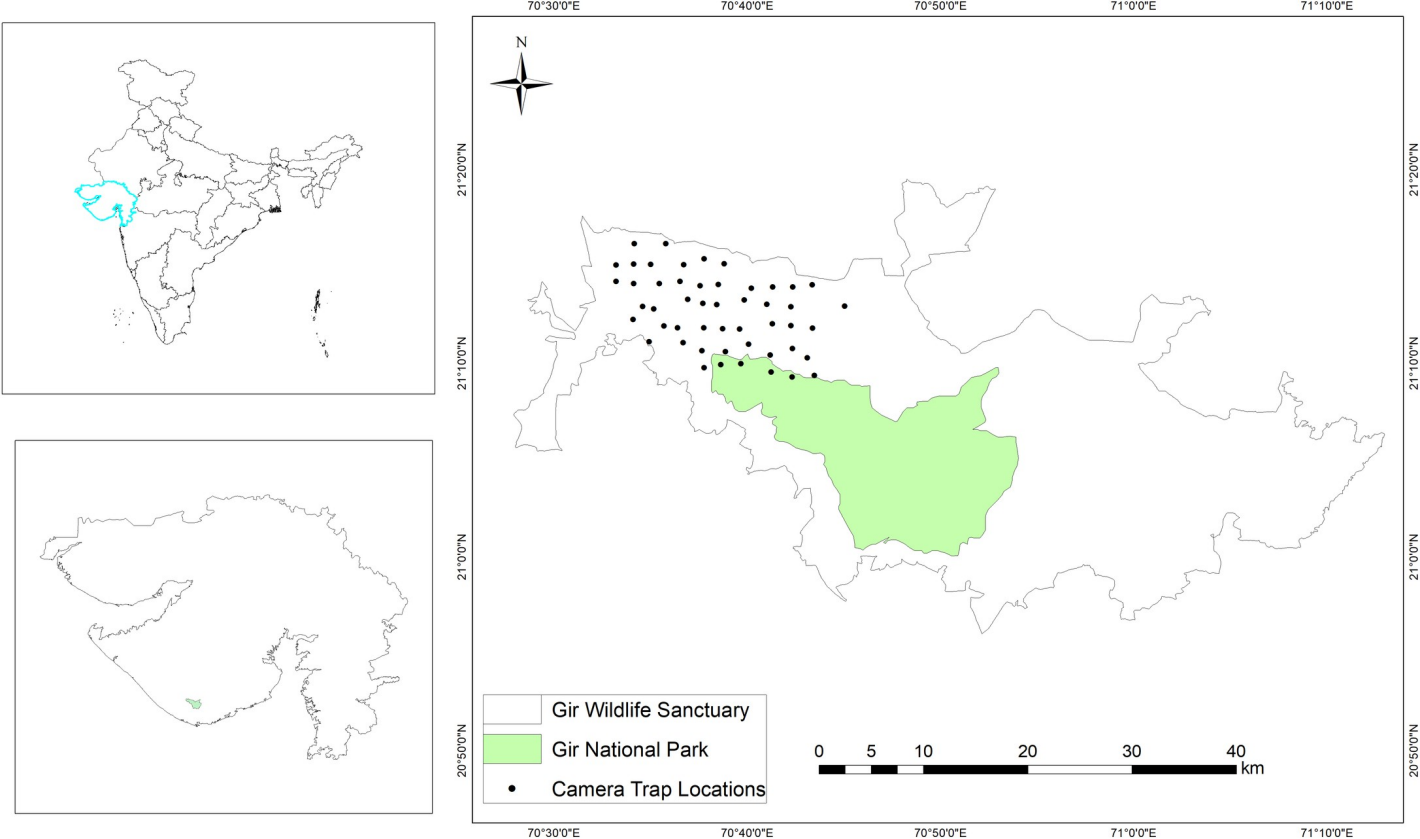
Map of the study area with locations of camera traps.

Camera trapping was used to fulfil the objectives of the present study. An area of 200 km^2^ was selected in the western part of Gir, representing major habitat types. This area was further divided into 50 grids of 4 km^2^ area each. In each grid, a pair of camera traps was placed, which remained active for 24 hours (Fig 1). Camera trapping was carried out from March 2017 to June 2017 in summer and from November 2017 to January 2017 in winter. Due to logistic constraints, camera traps were placed only at 10 sites in winter. They were placed along trails and roads, each fixed with a wooden block at 35 cm above the ground. Due to better availability of roads and trails network, camera traps were monitored thrice a week.

### Data analysis

Each camera trap capture was recognized as an independent event when the delay between the successive photo-captures at the same trap station was more than 30 minutes [32]. The time stamped on camera trap images was used in activity analysis. The circular statistic was used to describe the activity pattern, and Rayleigh’s test [33] to assess uniformity in the activity pattern of predators and their prey. We followed Linkie and Ridout [34] to determine the temporal overlap using R package *overlap* by calculating the coefficient of overlap [35] in R statistical and computing program (R Development Core Team 2018). The values of the coefficient of overlap vary between 0 (No overlap) to 1 (Complete overlap). Following the recommendation of Meredith and Ridout [35], the Dhat 4 coefficient of overlap was used for lower sample size that was more than 75, and Dhat 1 was used for lower sample size less than 75. We generated 10000 bootstrapping iterations to calculate 95% confidence intervals for activity overlap. Spearman rank correlation test [33] was used to assess the inter-predator and prey-predator temporal association between the percent hourly activity of predators and their prey.

To assess the space use patterns and habitat preferences of predators and their prey, the intensive study area was divided into four different habitats following Qureshi and Shah [36]. These include Teak-Acacia-Zizyphus, distributed in flat areas with less ground and canopy cover, Moist Mixed, including riverine habitat with undulating terrain and dense ground and canopy cover, Mixed habitat, distributed in flat and hilly slopes with dense ground and canopy cover, and Thorn habitat, distributed in flat areas with open ground and canopy cover. All the camera trap sites were classified in relation to the above defined habitat types. Percent use of each habitat by leopard, lion, and their prey was used to assess their space use pattern. Jacobs index was used to assess the habitat preference of predators and prey [37]. The values of Jacobs index range between +1 (Preference) to -1 (Avoidance). Hierarchal cluster analysis was used to assess inter-predator and prey-predator associations in habitat use. Cluster analysis was performed on the abundance of predators and prey in each habitat type using chi-square distance [38] and Pianka niche overlap index [39] to assess the spatial overlap. Pianka niche overlap index provides values between 0 and 1, with 0 indicating no overlap whereas 1 meaning total overlap. To assess the site-specific association between leopards and lions in relation to their prey, we first calculated relative abundance index (RAI) at the camera trap site by dividing the independent number of pictures by total trap nights at any particular site [32]. Thereafter, Spearman rank correlation test [33] was used between RAI of leopard and lion and their prey species at each site to assess inter predator and prey-predator associations [32].

## Results

### Sampling effort

Total camera trapping efforts resulted in getting 2003 trap nights; the mean trap night per site was (30.8 ± 1.8). Leopard had the highest independent photo captures (289) than the lion (153). Among prey species, chital had the highest independent photo captures (631), followed by sambar (101), wild pig (50), and nilgai (33).

### Activity pattern

**Fig 2:**
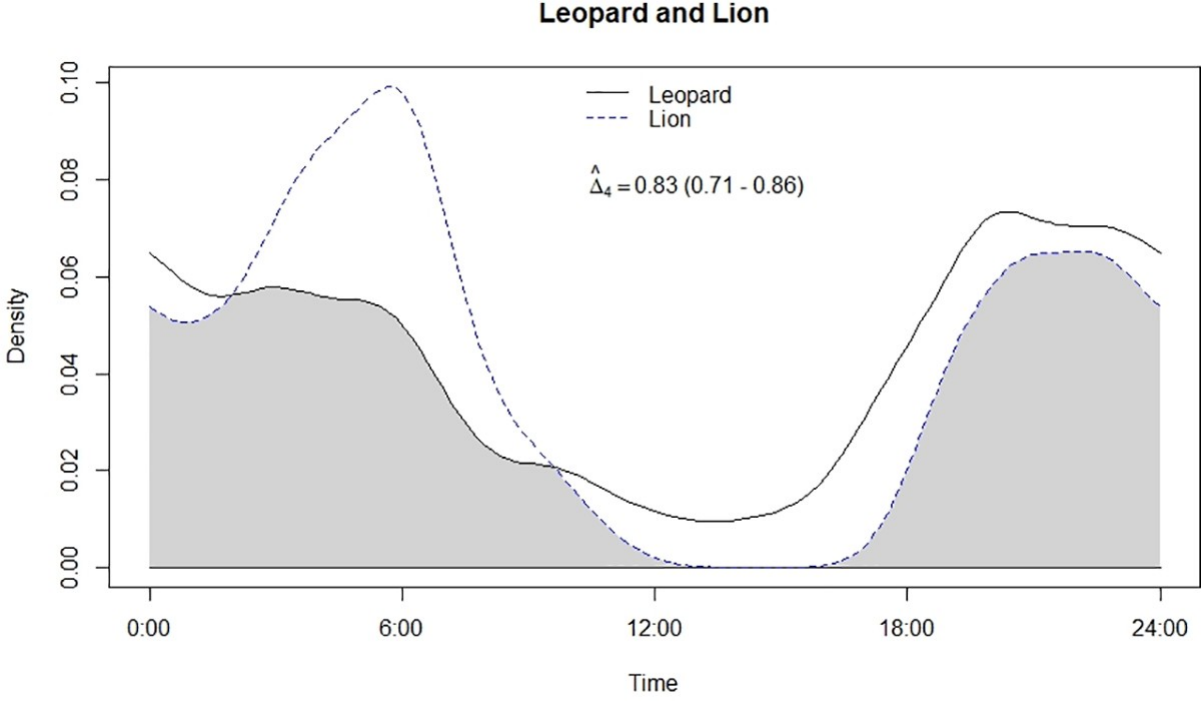
Temporal overlap between leopard and lion.

**Fig 3:**
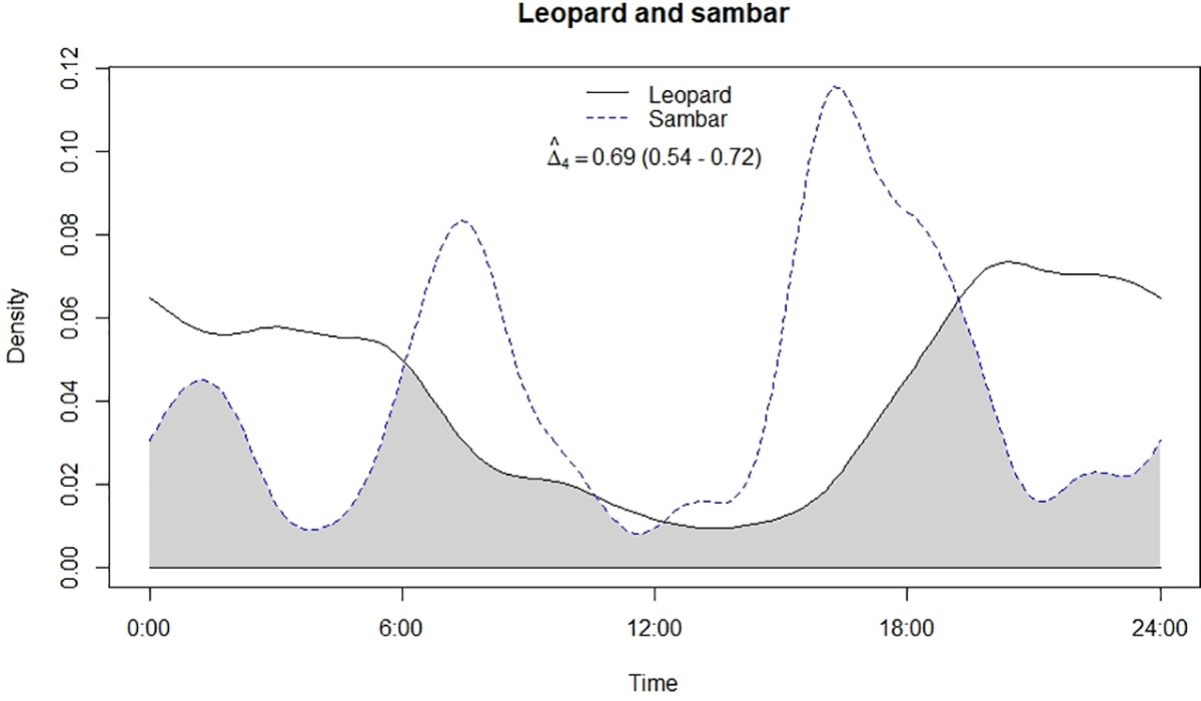
Temporal overlap between leopard and sambar.

**Fig 4:**
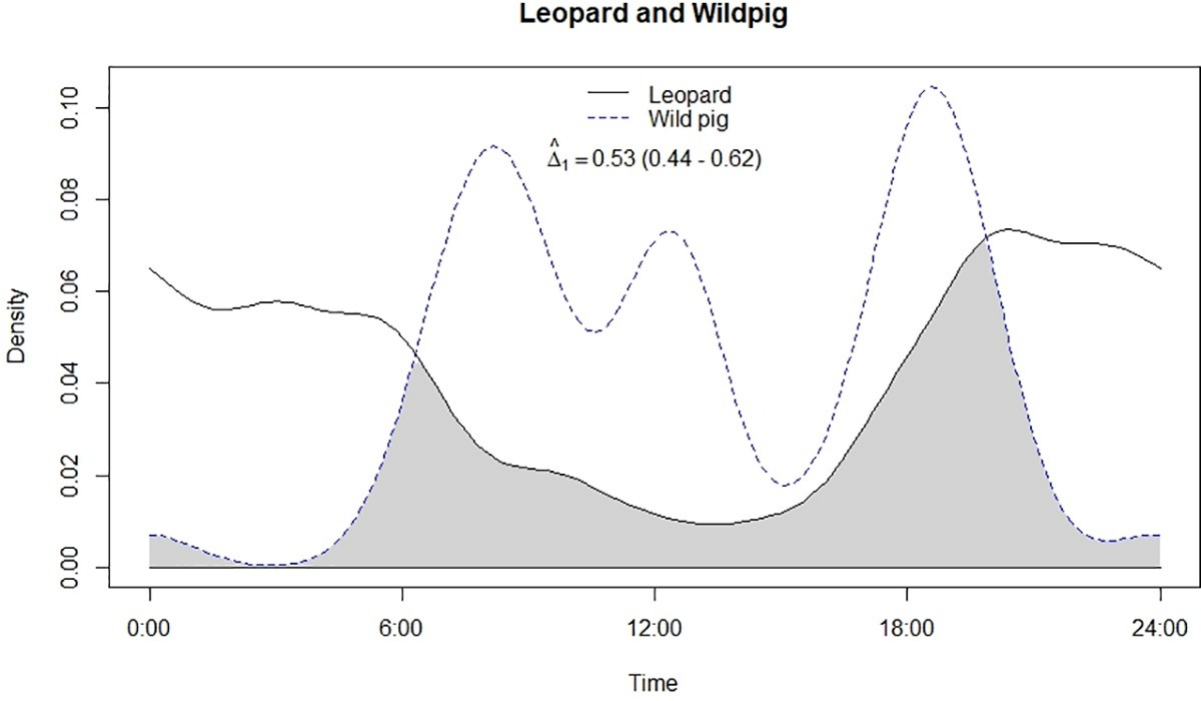
Temporal overlap between leopard and wild pig.

**Fig 5:**
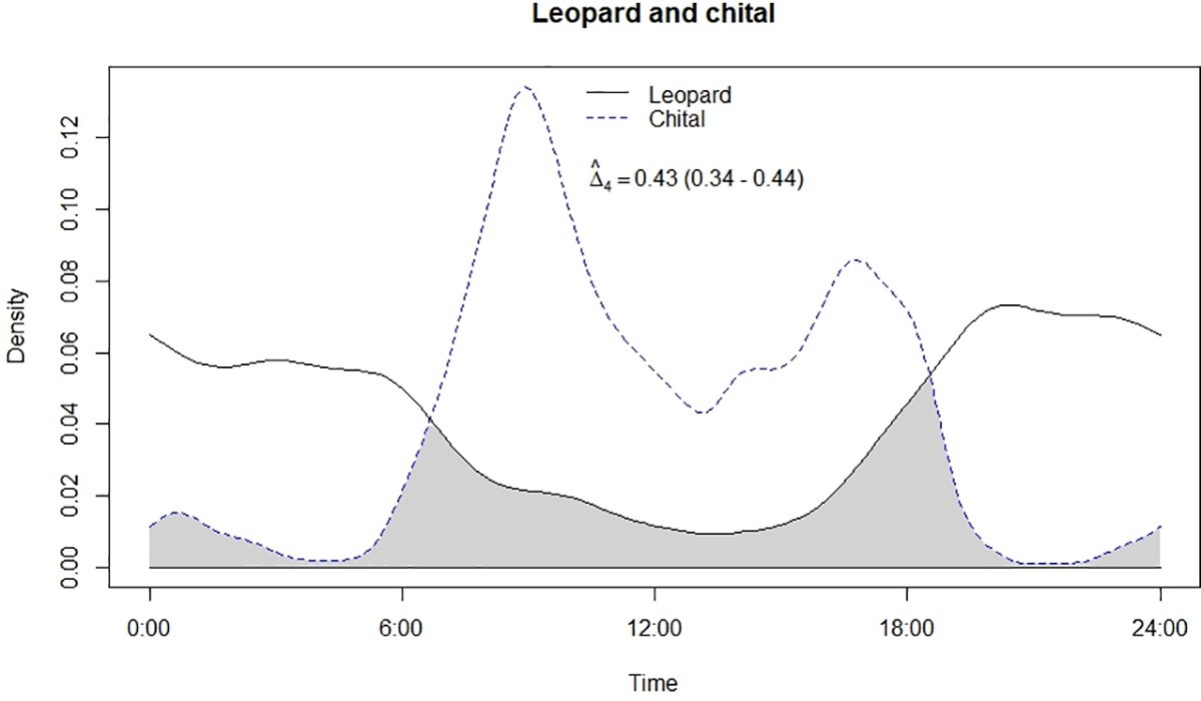
Temporal overlap between leopard and chital.

**Fig 6:**
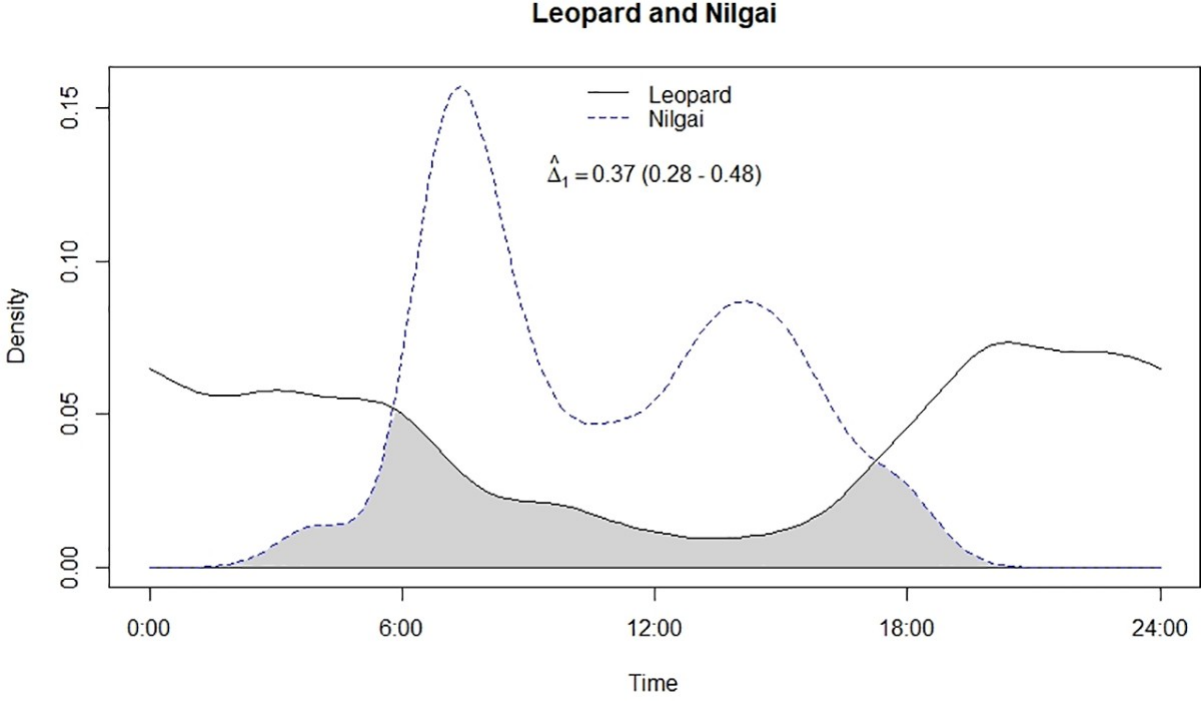
Temporal overlap between leopard and nilgai.

Both leopard and lion were crepuscular and nocturnal having a bimodal peak in activity (Fig 2). Mean activity of leopards was recorded during 23:11 ± 00:41 Hrs [95% confidence intervals (CI): 21:49–00:32 Hrs] (Table 1) with non-uniform activity (Z = 22.15, p<0.05). On the other hand, mean activity of lion was recorded during 01:20 ± 00:21 Hrs [95% confidence intervals (CI): 00:39–02:02 Hrs], also having non-uniform activity (Z = 51.6, p<0.05) like leopards. Among prey types, chital and nilgai were strictly diurnal while sambar and wild pig were crepuscular to nocturnal in activity (Figs 3–6). Mean activity of chital was 11:30 ± 00:27 Hrs [95% confidence intervals (CI): 10:36–12:23 Hrs] with non-uniform activity (Z = 30.34, p<0.05) and it was 11:04 ± 00:26 Hrs [95% confidence intervals (CI): 10:11–11:56 Hrs] for nilgai with non-uniform activity (Z = 30.44, p<0.05). Mean activity of sambar was during 15:17 ± 01:14 Hrs [95% confidence intervals (CI): 12:50–17:43 Hrs] with non-uniform activity (Z = 4.59, p<0.05) whereas for wild pig, mean activity was during 13:52 ± 00:55 Hrs [95% confidence intervals (CI): 12:04–15:41 Hrs], also with non-uniform activity (Z = 30.34, p<0.05).

**Table 1:** Circular statistics of activity pattern of leopard, lion and their prey in Gir.

Variable	Leopard	Lion	Chital	Sambar	Nilgai	Wild Pig
**Number of observations**	289	153	631	101	33	50
**Mean vector**	23:11	01:20	11:30	15:17	11:04	13:52
**Circular variance**	0.62	0.535	0.489	0.787	0.445	0.714
**Standard error of mean**	00:41	00:21	00:27	01:14	00:26	00:55
**95% confidence intervals**	21:49–00:32	00:39–02:02	10:36–12:23	12:50–17:43	10:11–11:56	12:04–15:41
**Rayleigh’s test**	14.08, p<0.001	51.6, p<0.05	30.34, p<0.05	Z = 4.59, p<0.05	Z = 30.44, p<0.05	Z = 30.34, p<0.05

### Temporal overlap and association in activity

**Fig 7:**
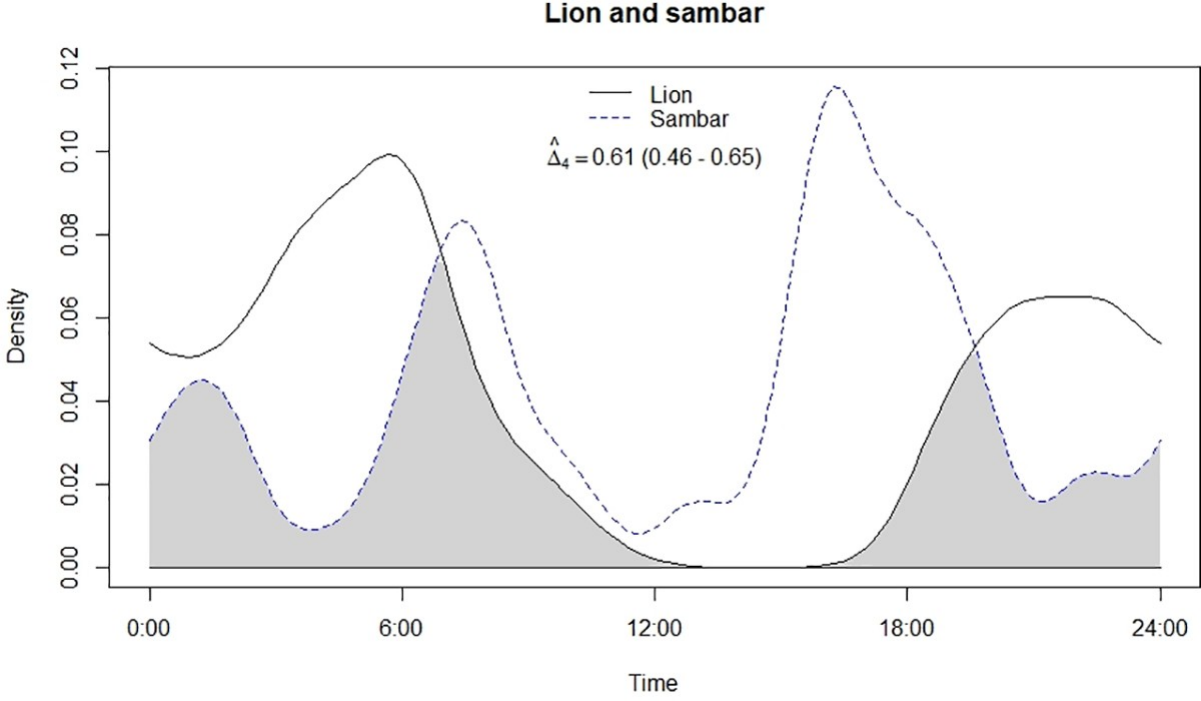
Temporal overlap between lion and sambar.

**Fig 8:**
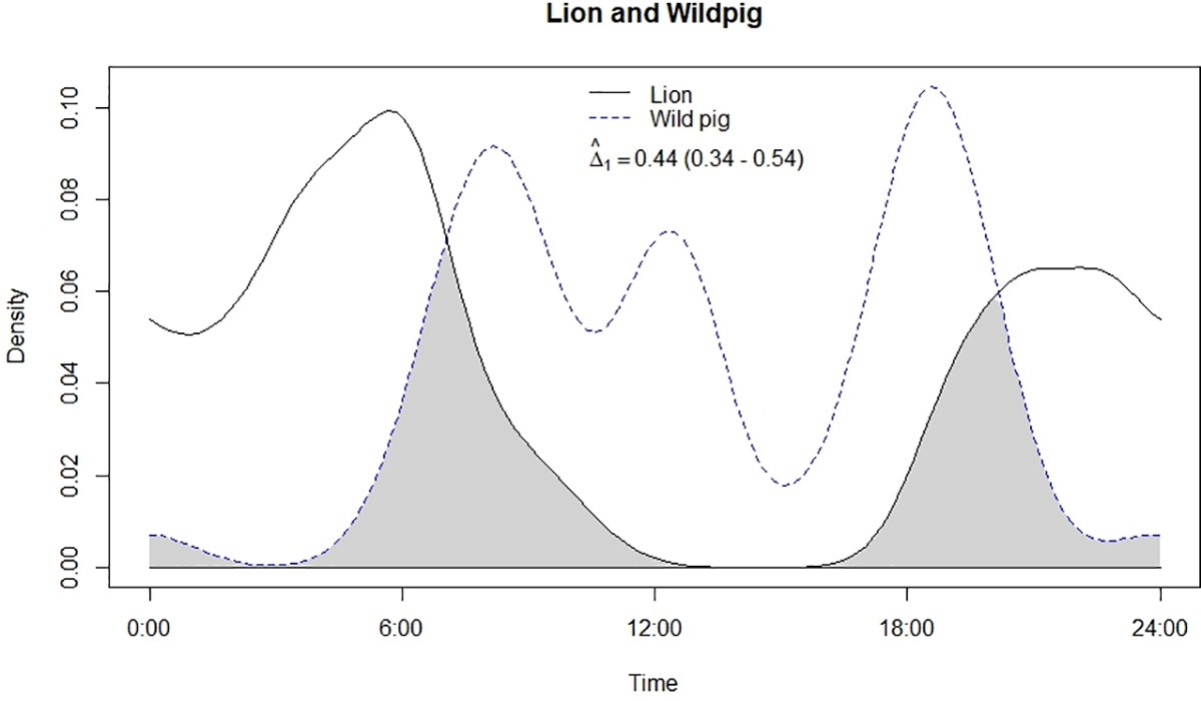
Temporal overlap between lion and wild pig.

**Fig 9:**
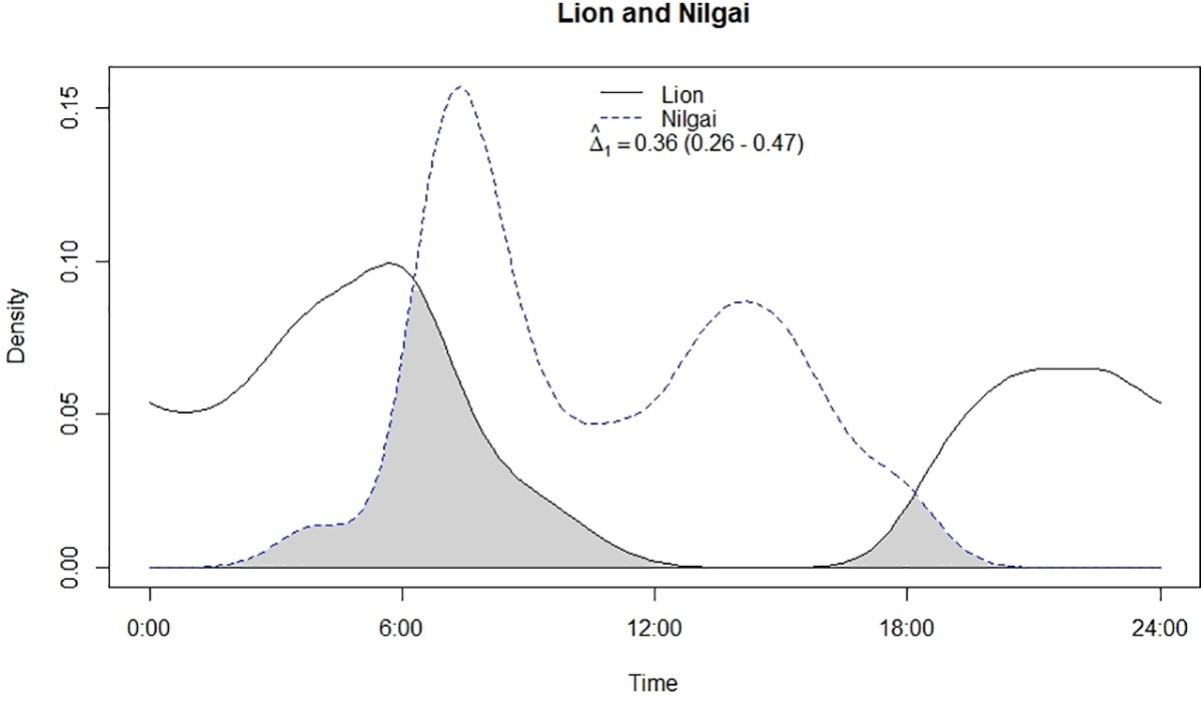
Temporal overlap between lion and nilgai.

**Fig 10:**
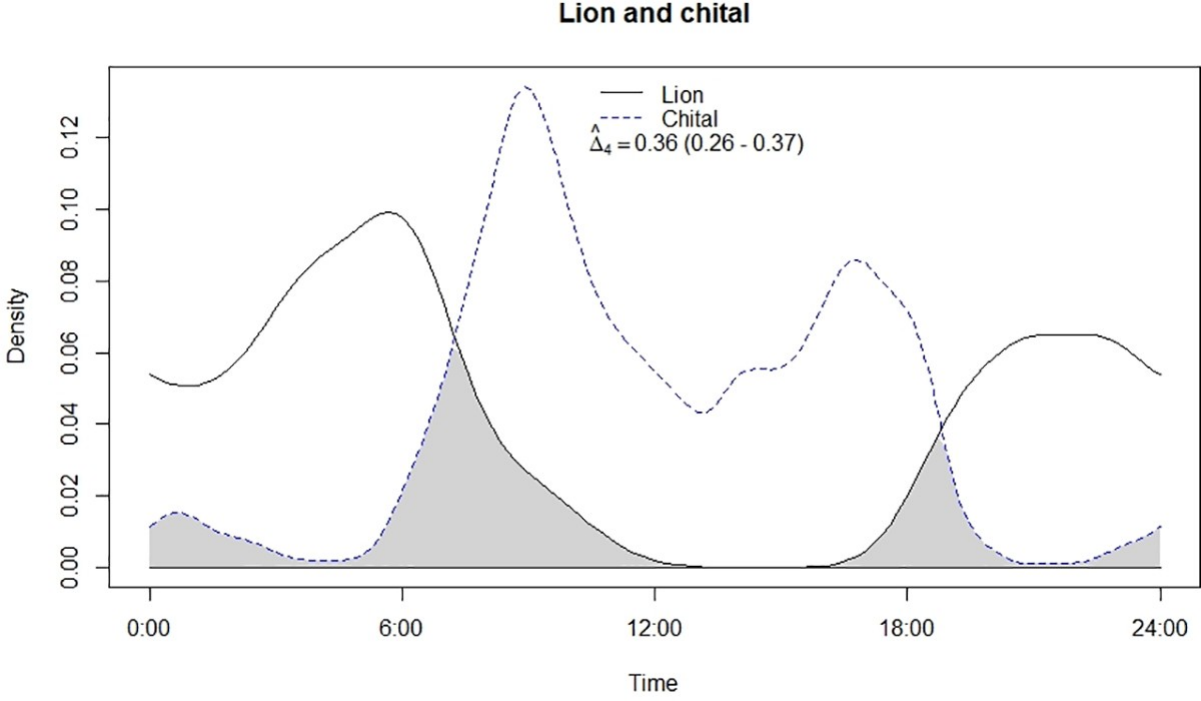
Temporal overlap between lion and chital.

Leopard and lion showed high temporal overlap (0.80; 95% CI: 0.72–0.86) (Table 2) besides having a strong positive association in their activity (r = 0.73, p<0.01) (Fig 2). In relation to prey, leopard showed highest temporal overlap with sambar (0.69; 95% CI: 0.54–0.72) (Fig 3) followed by wild pig (0.53; 95% CI: 0.44–0.62) (Fig 4), chital (0.43; 95% CI: 0.34–0.44) (Fig 5) and nilgai (0.37; 95% CI: 0.28–0.48) (Fig 6). Correlation analysis showed that leopard activity pattern was associated negatively with chital (r = -0.69, p<0.05) and nilgai (r = -0.44, p<0.05) and indicated no temporal association with sambar (r = 0.12, p>0.05) and wild pig (r = -0.1, p>0.05). Likewise, lion in relation to prey revealed highest temporal overlap with sambar (0.61; 95% CI: 0.46–0.65) (Fig 7) followed by wild pig (0.44; 95% CI: 0.34–0.54) (Fig 8), nilgai (0.36; 95% CI: 0.26–0.47) (Fig 9) and chital (0.36; 95% CI: 0.26–0.) (Fig 10). Correlation analysis showed that lion activity pattern was associated negatively with chital (r = -0.71, p<0.05) and nilgai (r = -0.63, p<0.05) and indicated no temporal association with sambar (r = -0.30, p>0.05) and wild pig (r = -0.1, p>0.05).

**Table 2:** Spatial and temporal overlap and association between leopard and lion and with their prey.

	Temporal overlap and association		Spatial overlap and association	
	Overlap (95%CI)	Correlation coefficient (r)	Overlap	Correlation coefficient (r)
**Leopard-lion**	80 (0.72–0.86)	0.73*	0.51	.041
**Leopard-Chital**	0.43 (0.34–0.44)	-0.69*	0.40	0.34*
**Leopard-Sambar**	0.69 (0.54–0.72)	0.12	0.42	0.23*
**Leopard-Nilgai**	0.37 (0.28–0.48)	-0.44*	0.32	0.16
**Leopard-Wild pig**	0.53 (0.44–0.62)	-0.1	0.26	0.11
**Lion-Chital**	0.36 (0.26–0)	-0.71*	0.42	.07
**Lion-Sambar**	0.61 (0.46–0.65)	-0.30	0.14	-0.53*
**Lion-Nilgai**	0.36 (0.26–0.47)	-0.63*	0.26	.09
**Lion-Wild pig**	0.44 (0.34–0.54)	-0.1	0.12	-0.28*

* p<0.05

### Space use pattern and habitat preferences

**Fig 11:**
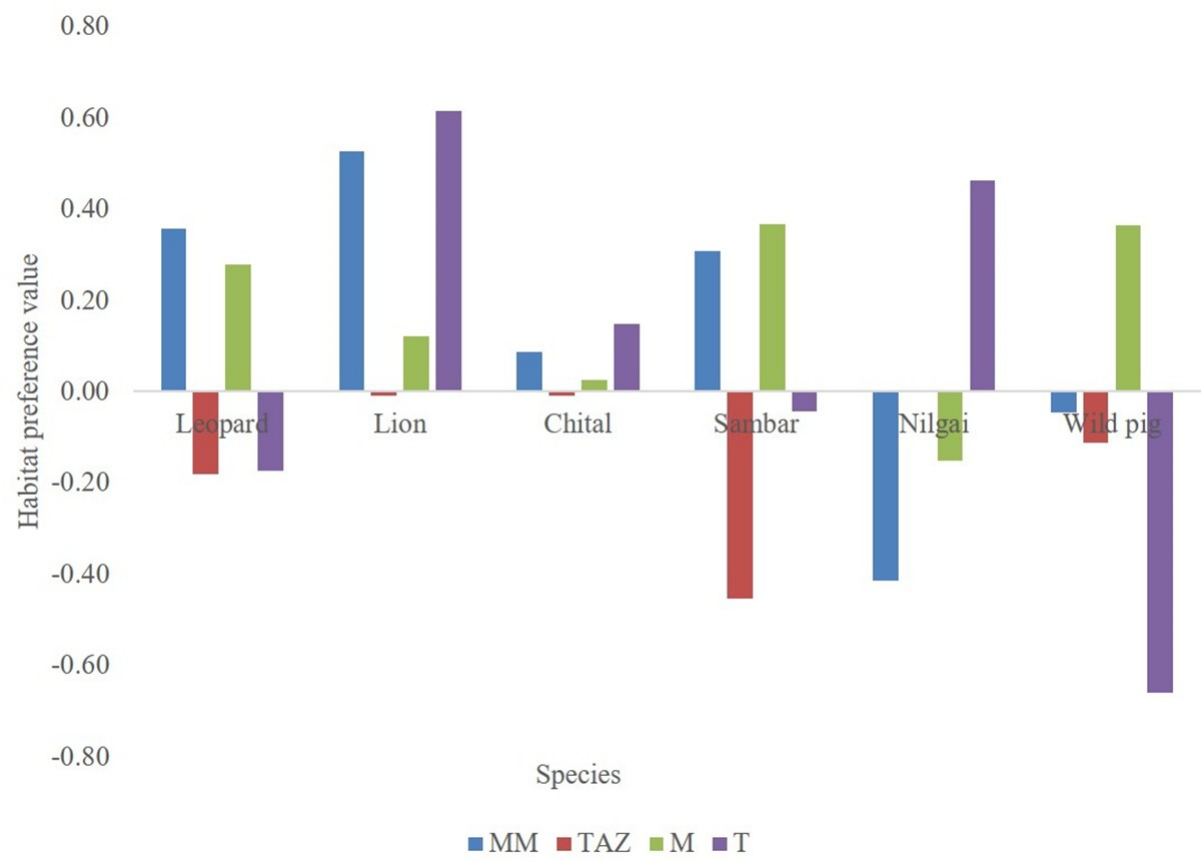
Habitat preference of leopard, lion and their prey.

Based on camera traps, Leopards used Mixed habitat maximum followed by Moist Mixed, Teak Acacia Zizyphus, and Thorn habitat (Table 3). Lions, instead used Moist Mixed habitat most followed by Teak Acacia Zizyphus, Thorn habitat, and Mixed habitat. Chital was found to use Teak Acacia Zizyphus habitat most often followed by Mixed, Moist Mixed, and Thorn habitat while sambar used Mixed habitat most followed by Moist Mixed, Teak Acacia Zizyphus and Thorn habitat. Nilgai used Teak Acacia Zizyphus habitat most often followed by Thorn, Mixed, and Moist Mixed habitat while wild pig used Mixed habitat mostly followed by Teak Acacia Zizyphus, Moist Mixed, and Thorn habitat. Habitat preference analysis showed that leopard preferred dense habitat, i.e., Moist Mixed and Mixed and avoided open habitats, namely Teak Acacia Zizyphus and Thorn. Lion preferred both dense and open habitats, i.e., Moist Mixed, Mixed and Thorn while avoided Teak Acacia Zizyphus habitat. Chital showed preference for Thorn, Moist Mixed, and Mixed habitat and avoided Teak Acacia Zizyphus habitat. Sambar showed preference for Moist Mixed and Mixed habitat and avoided Thorn and Teak Acacia Zizyphus habitat. Nilgai showed preference towards Thorn and Teak Acacia Zizyphus habitats and avoided Moist Mixed and Mixed habitat. Wild pig preferred Mixed habitat and avoided Moist Mixed, Teak Acacia Zizyphus, and Thorn habitats (Fig 11).

**Table 3:** Percent habitat used by leopard, lion and their prey in different habitat.

Habitat type	Leopard	Lion	Chital	Sambar	Nilgai	Wild pig
**Moist Mixed**	30	35.3	15.4	26.1	5.1	11.5
**Teak Acacia Zizyphus**	27.9	31.1	55.7	13	61.5	34.7
**Mixed**	37	13.6	19	53.2	12.8	52.1
**Thorn**	5	19.8	9.7	7.4	20.5	1.4

### Spatial overlap and association

**Fig 12:**
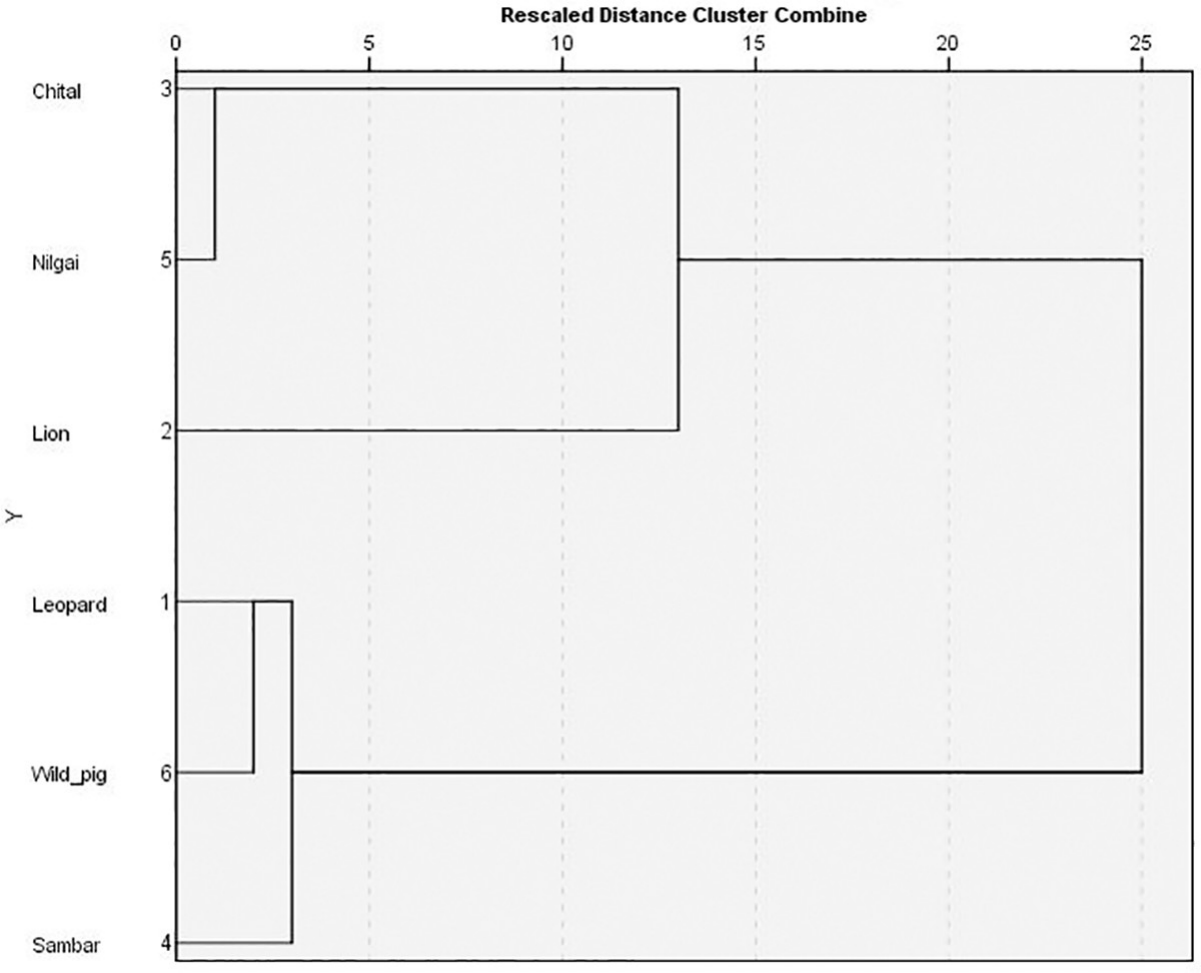
Dendrogram showing inter-predator and prey-predator association in habitat use.

Leopard and lion showed less spatial overlap (0.51) (Table 2) and they also indicated no association in space use (r = .041, p>0.05). In relation to prey, spatial overlap of leopard was highest with sambar (0.42) followed by chital (0.40), nilgai (0.32), and wild pig (0.26). Correlation analysis revealed that leopard was positively associated in space use with two of its key prey species, i.e., chital (r = 0.34, p<0.05) and sambar (r = 0.23, p<0.05) and did not show any association with nilgai (r = 0.16, p>0.05) and wild pig (r = 0.11, p>0.05). On the contrary, the lion in relation to prey showed highest spatial overlap with chital (0.42) followed by nilgai (0.26), sambar (0.14), and wild pig (0.12). Interestingly, correlation analysis showed negative spatial association of lion with two of its preferred and key prey species, i.e., sambar (r = -0.53, p<0.05) and wild pig (r = -0.28, p<0.05) and no association with chital (r = .07, p>0.05) and nilgai (r = .09, p>0.05). Cluster analysis revealed that the leopard was not associated with the lion in habitat use. Habitat use by leopard was associated with sambar and wild pig, while it was associated with chital and nilgai in case of lion (Fig 12).

## Discussion

We did not find any support for our hypothesis along the temporal axis, since overlap between leopards and lion is high and strong positive association exist in their activity pattern. Our findings support recent studies conducted on leopards and lions in the African ecosystem [20–21, 24] and between leopards and tigers [40–42] where leopards show high temporal overlap with lion and tiger. Karanth et al. [9] have found that high prey availability may restrict the activity of carnivores due to low searching and hunting efforts. The high prey densities (>50 km^2^) in Gir might lead to the limited activity of both the predators in certain hours of the diel as shown by strong positive correlation in activity pattern as a consequence of which there is a high temporal overlap between them. By remaining active at dawn and dusk, leopards seem to get benefitted from low light conditions. Low light conditions, on the other hand, create reliable conditions for ambush predators and also decrease the probability of their encounter with the dominant member of the guild, i.e., lion in this case.

Optimal foraging theory predicts that predators will synchronize their activity with their preferred prey to increase their encounter rates and hence optimize the hunting efficiency [43]. Sambar is the preferred prey of leopard as well as lion, while wild pig is the preferred prey species of lions in Gir [25]. Substantial temporal overlaps of leopard and lion with sambar and of the lion with wild pig might be a strategy by both the predators to increase their foraging success by remaining active during the same time when their preferred prey species are active. Chital is another key species of leopards and lions, but both the predators showed less temporal overlap and negative hourly relationship with chital. It is quite possible that chital predation may occur during night or crepuscular hours. High temporal overlap, along with association, also indicates the limited role of temporal partitioning in the coexistence of leopard and lion.

We found support for our hypothesis along the spatial axis since leopards overlap less in space and also there was no association in their space use. This less overlap and no spatial association can be discussed in the light of differential habitat use. Cluster analysis showed that the leopard was in a different cluster than the lion. The association of habitat use by leopard with sambar and wild pig indicates its affinity towards dense habitat and earlier studies have also found both prey dyad (sambar and wild pig) and leopard using dense habitat heavily [44–46]. But the lion association of habitat use with chital and nilgai indicates the use of open habitat more since both chital and nilgai are species that prefer open habitat [45]. This differential association in habitat use of lion and leopard may also result in low spatial overlap between them. Habitat preference analysis revealed that both predators prefer dense habitat (Moist and Moist Mixed), and substantial partitioning occurs in the use of open habitat (Thorn), which are avoided by leopards. Moist and Moist-mixed habitats among the habitat types in our study area have dense canopy and understory cover due to the distribution of rivulets and water bodies around [36]. This dense vegetation structure creates a kind of visibility hindrance; consequently, less interference encounters between leopard and lion. Recently, Rafiq et al. [47] have also reported that leopard and lion encounters occurred less in dense habitat due to visibility obstruction. Balme et al. [22] have also termed such habitats as “hideable habitat” for leopards, which act as a refuge for them to avoid a dominant member of the guild. Also, these habitats provide good ambush conditions for leopard and lion which in turn might be helpful in increasing their hunting success [44, 48]. Thorn habitat in our study area has less canopy cover and largely more open ground cover [45]. Less canopy cover and open understory can increase the chances of encounters between leopard and lion. Du preez et al. [49] have also found that leopards avoid lions to maximum in open habitat. High visibility along with less vegetation cover may not be very promising for leopards to prefer thorn habitat resulting thereby in differential preference for this habitat by leopards and lions. Among prey species, leopard showed highest spatial overlap and significant spatial associations with two of its key prey species; sambar and chital. Both chital and sambar are the key prey species of leopards [25]. The highest overlap with chital and sambar along with positive spatial association might be a strategy to increase their encounters during prey search and eventually foraging success. However, lion showed less overlap and negative spatial association with two of its preferred prey species, i.e., wild pig and sambar. Habitat is one of the critical determinants in prey-predator relationship [50]. Negative spatial association between lion and prey dyad (sambar and wild pig) might be a result of differential habitat preferences and site use by them. It is probably due to the fact that sambar has a preference for hilly and dense habitat in Gir [45] and wild pig also used the habitats having dense understory in our study area (personal observation). Lions in Gir preferred flatter terrain [51]. Moreover, dense understory sites which are highly used by sambar and wild pig might cause hindrance in the movement for lions. This differential use of habitat and terrain may result in very less spatial overlap and negative spatial association between lion and the prey dyad (sambar and wild pig). However, the high spatial overlap of the lion with chital and nilgai as compared to the other prey dyad (wild pig and sambar) is possibly due to preference of lion and the prey dyad (chital and nilgai) for flatter terrain [45, 51].

We found niche complementary [5] between leopard and lion, which involves a high temporal overlap but low spatial overlap and hence space playing an important role in resource partitioning and coexistence of leopard and lion in Gir. Structural habitat heterogeneity (closed vs. Open) in Gir is a critical determinant of spatial partitioning between leopard and lion and hence crucial for coexistence of both the predators. Future change in habitat structure of Gir could have severe implications on coexistence of leopard and lion since both exist at very high densities. Since the scale of our study is limited to specific sites, there is an urgent need to assess how these two large predators coexist at a larger scale, such as home range.

## Supporting information

S1 File (DOCX)

S1 Data (XLSX)

S2 Data (XLSX)

S3 Data (XLSX)

S4 Data (DOCX)
