# Supplementary material for: Spatio-temporal partitioning and coexistence between leopard (Panthera pardus fusca) and Asiatic lion (Panthera leo persica) in Gir protected area, Gujarat, India
Source: PLoS One. 2020 Mar 11;15(3):e0229045. doi: 10.1371/journal.pone.0229045 (PMC7065753; doi:10.1371/journal.pone.0229045)
Supplement: S1 File — (DOCX) [file pone.0229045.s001.docx]

Manuscript edited by:

Dr. Satish Kumar, Associate Professor, Department of wildlife sciences, Aligarh Muslim University, Aligarh, India

Email id: satish_amu@yahoo.com

***Spatio-temporal partitioning and coexistence between leopard (Panthera pardus fusca) and Asiatic lion (Panthera leo persica) in Gir protected area, Gujarat, India***

Rohit Chaudhary^1*^, Nazneen Zehra^1^, Azra Musavi^2^ and Jamal Ahmad Khan^1^

^1^ Department of Wildlife Sciences, Aligarh Muslim University, Aligarh, India

^2^Centre for Woman’s studies, Aligarh Muslim University, Aligarh, India

^*^ Corresponding author

Email: rchaudhary259@gmail.com

**Abstract**

Time and space are essential niche dimensions along which species tend to coexist. We assessed spatiotemporal resource partitioning between leopards and lions and hypothesized the differential use of spatiotemporal resources by leopards with respect to lions. We used a systematic camera trap survey to collect the data at 50 sites. The data was analyzed using overlap indices, and non-parametric test statistic was used to analyze the spatiotemporal associations. Leopard and lion were mainly crepuscular and nocturnal in their activity pattern. They did not segregate temporally and showed substantially high overlap and strong temporal association. Leopard segregates with lion spatially by overlapping less and showing no association in space use at specific camera trap sites. Leopards showed preference for dense habitats, while the lion preferred both dense and open habitats. Leopard showed moderate-overlap and positive association with key prey species, i.e., chital and sambar. Lion, however showed low site-specific overlap and negative association with its crucial prey species, i.e., sambar and wild pig. We conclude that site-specific spatial partitioning along with differential affinities for habitat is helping leopards to partition their food resources with lions and hence facilitate coexistence of leopards with lions in Gir forest.

Keywords: Activity pattern, Habitat, Overlap, Coexistence, Predators

**Introduction**

Interspecific competition is among the major forces responsible for shaping the community structure and functioning [1]. Interspecific competition could be interference, where the dominant member in the guild can directly affect a subordinate member of the guild or exploitative where a dominant member of the guild may reduce resource availability without interacting directly with a subordinate member of the guild [2]. Gauss's exclusion principle states that species with similar resource requirements cannot coexist, and one of the sympatric species will be excluded [3,4]. Coexistence between sympatric species is possible when they partition themselves along the spatial, temporal, or dietary axis to reduce the ecological overlap [5,6]. Therefore, resource partitioning is among the key processes responsible for the coexistence of sympatric species.

Large carnivores owing to their predatory behavior and being at the top position can affect community structure disproportionately [7]. Due to their morphological similarities and similar resource requirement, sympatric large carnivores often result in higher overlap in resource use and could end up in competition. Competitive interactions among large carnivores are mostly asymmetrical when a dominant member of the guild can affect the abundance, distribution, and resource use patterns of the subordinate members of the guild [8]. Due to the substantial habitat loss, much of the carnivore population now exists in fragmented habitats, where the interspecific interactions may be intense [9].

Therefore, current competition in large carnivore guild cannot be ignored, and hence resource partitioning studies play an essential role in understanding how subordinate members of the guild partition their resources and coexist. Resource partitioning among large carnivores can be achieved through a variety of ecological processes such as dietary partitioning [10] and spatiotemporal partitioning [9,11]. Recent studies have also illustrated the importance of adjustment in fine space use vis-à-vis time between members of the carnivore guild to facilitate the resource partitioning. Such fine adjustment includes avoidance of risky habitat type where the encounter with a dominant competitor is more [12], avoiding hourly activity of dominant member [13] and avoidance of area with high temporal activity [14].

Common Leopard (*Panthera pardus fusca*) and Asiatic lion (*Panthera leo persica*) are two large carnivores that occur sympatrically in Gir Wildlife Sanctuary and National Park (Hereafter Gir). This Guild provides a unique opportunity to assess resource partitioning due to the existence of the sole population of Asiatic lions in Gir. There have been studies on resource partitioning and coexistence among lions and leopards from the African ecosystem [15-24], but no study has been conducted yet regarding how leopard and Asiatic lion segregate resources and coexist. Zehra et al. [25] found that leopard and lion overlap very high (>90%) in their dietary habits, which indicate the limited role of the diet in defining resource partitioning between these two predators. However, space use and activity pattern may possibly play an important role in resource partitioning between these two predators. Further, Gir harbours high densities of both leopards (19.8 individual/ 100 km2) [26] and lions (15 individuals / 100 km2) [27]. At such high densities, there is a high probability of direct encounters between leopards and lions. Spatiotemporal partitioning is among the one of the key processes for predator-predator coexistence by minimizing the direct encounters [28]. Hence understanding how leopard and lion partition their space and time is important to understand their coexistence .

The goal of the present study was to understand spatiotemporal resource partitioning between the leopard and Asiatic lion. We asked two research questions in study i) How do leopards partition resources spatiotemporally with lions and coexist? ii) Between space and time, which axis contributes more to the coexistence of leopard with the lion? Due to smaller body size, leopards are subordinate to lions in Gir, and therefore we hypothesize that leopards will use space and time differently than lions in Gir. Prediction for this hypothesis was that leopard would show less spatial and temporal overlap, differential habitat preference to lions, and also show no association in space use and activity pattern with lions. Further, Lovari et al. [29] stated that tigers and leopards do not segregate along single niche dimensions, and partitioning along the three major niche dimensions (time, space, and food) contributes to their coexistence.

Therefore, our second hypothesis was that both space and time will contribute to the coexistence of leopard and lion. The major objectives of the study were 1) To assess the pattern of space and time use by leopard and lion 2) To assess overlap and partition of space and time by leopards and lions.

**Materials and Methods**

**Study area**

The present study was carried out in Gir Wildlife Sanctuary and National Park, situated in the Semi-arid biogeographic zone of India. Forest type in Gir comes under very dry teak forest [30] [For more details, see 31].

Fig 1. Map of the study area with locations of camera traps

**Data collection**

Camera trapping was used to fulfil the objectives of the present study. An area of 200 km^2^ was selected in the western part of Gir, representing major habitat types. This area was further divided into 50 grids of 4 km^2^ area each. In each grid, a pair of camera traps was placed, which remained active for 24 hours (Fig 1). Camera trapping was carried out from March 2017 to June 2017 in summer and from November 2017 to January 2017 in winter. Due to logistic constraints, camera traps were placed only at 10 sites in winter. They were placed along trails and roads, each fixed with a wooden block at 35 cm above the ground. Due to the better availability of roads and trail network, camera traps were monitored thrice a week.

**Data analysis**

Each camera trap capture was recognized as an independent event when the delay between the successive photo-captures at the same trap station was more than 30 minutes [32]. The time stamped on camera trap images was used in activity analysis. The circular statistic was used to describe the activity pattern, and Rayleigh’s test [33] to assess uniformity in the activity pattern of predators and their prey. We followed Linkie and Ridout [34] to determine the temporal overlap using R package *overlap*by calculating the coefficient of overlap [35] in R statistical and computing program (R Development Core Team 2018). The values of the coefficient of overlap vary between 0 (No overlap) to 1 (Complete overlap). Following the recommendation of Meredith and Ridout [35], the Dhat 4 coefficient of overlap was used for lower sample size that was more than 75, and Dhat 1 was used for lower sample size less than 75. We generated 10000 bootstrapping iterations to calculate 95% confidence intervals for activity overlap. Spearman rank correlation test [33] was used to assess the inter-predator and prey-predator temporal association between the percent hourly activity of predators and their prey.

To assess the space use patterns and habitat preferences of predators and their prey, the intensive study area was divided into four different habitats following Qureshi and Shah [36]. These include Teak-Acacia-Zizyphus (TAZ), distributed in flat areas with less ground and canopy cover, Moist-mixed (MM), including riverine habitat with undulating terrain and dense ground and canopy cover, Mix (M) habitat, distributed in flat and hilly slopes with dense ground and canopy cover, and Thorn woodland (T), distributed in flat areas with open ground and canopy cover. All the camera trap sites were classified in relation to the above-defined habitat types. Percent use of each habitat by leopard, lion, and their prey was used to assess their space use pattern. Jacobs index was used to assess the habitat preference of predators and prey [37]. The values of Jacobs index range between +1 (Preference) to -1 (Avoidance). Hierarchal cluster analysis was used to assess inter-predator and prey-predator associations in habitat use. Cluster analysis was conducted on the abundance of predators and prey in each habitat using chi-square distance [38] and Pianka niche overlap index [39] to assess the spatial overlap. Pianka niche overlap index provides values between 0 to 1, 0 meaning no overlap and 1 meaning total overlap. To assess the site-specific association between leopards and lions in relation to their prey, we first calculated relative abundance index (RAI) at the camera trap site by dividing the independent number of pictures by total trap nights at any particular site [32]. Thereafter, Spearman rank correlation test [33] was used between RAI of leopard and lion and their prey species at each site to assess inter predator and prey-predator associations [32].

**Results**

**Sampling effort**

Total camera trapping efforts resulted in getting 2003 trap nights; the mean trap night per site was (30.8 ± 1.8). Leopard had the highest independent photo captures (289) than the lion (153). Among prey species, chital had the highest independent photo captures (631), followed by sambar (101), wild pig (50), and nilgai (33).

**Activity pattern**

Both leopard and lion were crepuscular and nocturnal having a bimodal peak in activity (Fig 2 ). Mean activity of leopards was recorded during 23:11 ± 00:41 Hrs [95% confidence intervals (CI): 21:49-00:32 Hrs] (Table 1) with non-uniform activity (Z=22.15, p<0.05). On the other hand, mean activity of lion was recorded during 01:20 ± 00:21 Hrs [95% confidence intervals (CI): 00:39-02:02 Hrs], also having non-uniform activity (Z=51.6, p<0.05) like leopards. Among prey, chital and nilgai were strictly diurnal while sambar and wild pig were crepuscular to nocturnal in activity (Fig 3-6 ). Mean activity of chital was during 11:30 ± 00:27 Hrs [95% confidence intervals (CI): 10:36-12:23 Hrs] with non-uniform activity (Z=30.34, p<0.05) and it was 11:04 ± 00:26 Hrs [95% confidence intervals (CI): 10:11-11:56 Hrs] for nilgai with non-uniform activity (Z=30.44, p<0.05). Mean activity of sambar was during 15:17 ± 01:14 Hrs [95% confidence intervals (CI): 12:50-17:43 Hrs] with non-uniform activity (Z=4.59, p<0.05) whereas for wild pig, mean activity was during 13:52 ± 00:55 Hrs [95% confidence intervals (CI): 12:04-15:41 Hrs], also with non-uniform activity (Z=30.34, p<0.05).

Table. 1. Circular statistics of activity pattern of leopard, lion and their prey in Gir

**Temporal overlap and association in activity**

Leopard and lion showed high temporal overlap (0.80; 95% CI: 0.72-0.86) (Table 2) besides having a strong positive association in their activity (r=0.73, p<0.01) (Fig 2 ). In relation to prey, leopard showed highest temporal overlap with sambar (0.69; 95% CI: 0.54-0.72) (Fig 3 ) followed by wild pig (0.53; 95% CI: 0.44-0.62) (Fig 4 ), chital (0.43; 95% CI: 0.34-0.44) (Fig. 5 ) and nilgai (0.37; 95% CI: 0.28-0.48) (Fig 6 ). Correlation analysis showed that leopard was associated negatively with chital (r=-0.69, p<0.05) and nilgai (r=-0.44, p<0.05) and indicated no association with sambar (r=0.12, p>0.05) and wild pig (r=-0.1, p>0.05). In relation to prey, lion revealed highest temporal overlap with sambar (0.61; 95% CI: 0.46-0.65) (Fig 7 ) followed by wild pig (0.44; 95% CI: 0.34-0.54) (Fig 8 ), nilgai (0.36; 95% CI: 0.26-0.47) (Fig 9 ) and chital (0.36; 95% CI: 0.26-0.) (Fig 10 ). Correlation analysis showed that lion was associated negatively with chital (r=-0.71, p<0.05) and nilgai (r=-0.63, p<0.05) and indicated no temporal association with sambar (r=-0.30, p>0.05) and wild boar (r=-0.1, p>0.05).

Fig 2. Temporal overlap between leopard and lion

Fig 3. Temporal overlap between leopard and sambar

Fig 4. Temporal overlap between leopard and wild pig

Fig 5. Temporal overlap between leopard and chital

Fig 6. Temporal overlap between leopard and nilgai

Fig 7. Temporal overlap between lion and sambar

Fig 8. Temporal overlap between lion and wild pig

Fig 9. Temporal overlap between lion and nilgai

Fig 10. Temporal overlap between lion and chital

Table 2. Spatial and temporal overlap and association between leopard and lion and with their prey

**Space use pattern and habitat preferences**

Based on camera traps, Leopards used M (Mixed) habitat maximum followed by MM, TAZ, and TW (Table 3). Lions, instead used MM habitat most followed by TAZ, T (Thorn Woodland), and M. Chital was found to use TAZ habitat most often followed by Mixed, MM, and Thorn Woodland while sambar used Mixed habitat most followed by MM, TAZ and Thorn Woodland. Nilgai used TAZ habitat most often followed by Thorn Woodland, Mixed, and MM forest types while Wild pig used Mixed habitat mostly followed by TAZ, MM, and TW. Habitat preference analysis showed that leopard preferred dense habitat, i.e., MM and M while avoiding open habitats, namely TAZ and Thorn Woodland. Lion preferred both dense and open habitats, i.e., MM, M and T while avoided TAZ. But chital showed preference towards T, MM, and M while avoided TAZ habitat. Sambar indicated a strong preference for MM and M and avoided T and TAZ habitat types. Nilgai showed preference towards T and TAZ habitats and avoiding MM and M. Wild pig showed a preference towards Mixed habitat and avoided MM, TAZ, and T habitats (Fig 11 ).

Habitat preference analysis showed that leopard preferred dense MM and Mixed habitat and avoided TAZ and Thorn habitat whereas Lion preferred MM, M, and Thorn Woodland and avoided TAZ habitat. Among prey species, chital indicated preference towards Thorn Woodland, MM, and Mixed habitat types and avoided TAZ habitat. Sambar was found to have strong preference for MM and Mixed habitat but avoided Thorn Woodland and TAZ habitat. But nilgai showed preference towards Thorn Woodland and TAZ habitats and avoided MM and Mixed habitat types whereas Wild pig preferred Mixed habitat and avoided MM, TAZ, and Thorn Woodland habitats (Fig 17).

Table 3 Percent habitat used by leopard, lion and their prey in different habitat

Fig 11 Habitat preference of leopard, lion and their prey

**Spatial overlap and association**

Leopard and lion showed less spatial overlap (0.51) (Table 2) and they also indicated no association in space use (r=.041, p>0.05). *In relation to*prey, spatial overlap of leopard was highest with sambar (0.42) followed by chital (0.40), nilgai (0.32), and wild boar (0.26). Correlation analysis revealed that leopard was positively associated in space use with two of its key prey species, i.e., chital (r=0.34, p<0.05) and sambar (r=0.23, p<0.05) and did not show any association with nilgai (r=0.16, p>0.05) and wild boar (r=0.11, p>0.05). On the contrary, the lion *in relation to* prey indicated highest spatial overlap with chital (0.42) followed by nilgai (0.26), sambar (0.14), and wild boar (0.12). Interestingly, correlation analysis showed negative spatial association of lion with two of its preferred and key prey species, i.e., sambar (r=-0.53, p<0.05) and wild boar (r=-0.28, p<0.05) and no association with chital (r=.07, p>0.05) and nilgai (r=.09, p>0.05). Cluster analysis revealed that the leopard was not associated with the lion in habitat use. Habitat use by leopard was associated with sambar and wild boar, while it was associated with chital and nilgai in case of lion (Figure 12 ).

Fig 12. . Dendrogram showing inter-predator and prey-predator association in habitat use

**Discussion**

We did not find any support for our hypothesis along the temporal axis, since and leopards overlap high and show strong positive association in activity pattern with lions**.** Our findings support recent studies conducted on leopards and lions in the African ecosystem [20-21, 24] and between leopards and tigers [40-42] where leopards show high temporal overlap with lion and tiger. Karanth et al. [9] have found that high prey availability may restrict the activity of carnivores due to low searching and hunting efforts. The high prey densities (>50 km^2^) in Gir might lead to the restricted activity of both the predators due to less search efforts and hence high temporal overlap. By remaining active at dawn and dusk, leopards seem to get benefitted from low light conditions. Low light conditions, on the one hand, create reliable conditions for ambush predators and also decrease the probability of encounter with the dominant member of the guild, i.e., lion in this case.

Optimal foraging theory predicts that predators will synchronize their activity with their preferred prey to increase their encounter rates and hence optimize the hunting efficiency [43]. Sambar is the preferred prey of leopard as well as lion, while wild boar is the preferred prey species of lions in Gir [25]. Substantial temporal overlaps of leopard and lion with sambar and of the lion with wild boar might be a strategy by both the predators to increase their foraging success by remaining active during the same time when their preferred prey species are active. Chital is another key species of leopards and lions, but both the predators showed less temporal overlap and negative hourly relationship with chital. It is quite possible chital predation may occur during night or crepuscular hours. High temporal overlap, along with association, also indicates the limited role of temporal partitioning in the coexistence of leopard and lion.

We found support for our hypothesis along the spatial axis since leopards overlap less in space and also there was no association in their space use. This less overlap and no spatial association can be discussed in the light of differential habitat use. Cluster analysis disclosed that the leopard was in a different cluster than the lion. The association of habitat use by leopard with sambar and wild boar indicates its affinity towards dense habitat since earlier studies also found both prey dyad (sambar and wild pig) and leopard use dense habitat heavily [44-46]. But the lion association of habitat use with chital and nilgai indicates the use of open habitat more since both chital and nilgai are species that prefer open habitat [45]. This differential association in habitat use of lion and leopard may also result in low spatial overlap between them. Habitat preference analysis revealed that both the predators prefer dense habitat (M and MM), and much of the partitioning occur in the use of open habitat (T), which are avoided by leopards. Moist and Moist-mixed habitats among the habitat types in our study area have dense canopy and understory cover due to the distribution of rivulets and water bodies around [36]. This dense vegetation structure creates a kind of visibility hindrance; consequently, less interference encounters between leopard and lion. Recently, Rafiq et al. [47] have also reported that leopard and lion encounters occurred less in dense habitat due to visibility obstruction. Balme et al. [22] have also termed such habitats as “hideable habitat” for leopards, which act as a refuge for them to avoid a dominant member of the guild. Also, these habitats provide good ambush conditions for prey concealment, which might be helpful in the increase in hunting success of leopard and lion. [44, 48]. Thorn woodland habitat in our study area has less canopy cover having open ground cover largely [45]. Less canopy and open understory can increase the chances of encounters between leopard and lion. Du preez et al. [49] have also found that leopards avoid lions to maximum in open habitat. High visibility along with less vegetation cover might do not allow leopards to prefer Thorn Woodland habitat resulting thereby in differential preference for this habitat by leopards and lions. Among prey species, leopard showed the highest temporal overlap and significant spatial associations with two of its key prey species; sambar and chital. Both chital and leopard are the key prey species of leopards [25]. The highest overlap with chital and sambar along with positive spatial association might be a strategy to increase their encounters during prey search and eventually foraging success. However, lion showed less overlap and negative spatial association with two of its preferred prey species, i.e., wild boar and sambar. Habitat is one of the critical determinants in prey-predator relationship [50]. Negative spatial association between lion and prey dyad (sambar and wild boar) might be a result of differential habitat preferences and site use by them. It is probably due to the fact that Sambar has a preference for hilly and dense habitat in Gir [45] and wild boar also used the habitats having dense understory in our study area (personal observation). Lions in Gir preferred flatter terrain [51]. Moreover, dense understory sites which are highly used by sambar and wild boar might cause hindrance in the movement for lions. This differential use of habitat and terrain may result in very less spatial overlap and negative spatial association between lion and the prey dyad (sambar and wild pig). However, the high spatial overlap of the lion with chital and nilgai as compared to the other prey dyad (wild boar and sambar) is possibly due to preference of lion and the prey dyad (chital and nilgai) for flatter terrain [45, 51].

We found niche complementary [5] between leopard and lion, which involves a high temporal overlap and low spatial overlap and hence space playing an important role in resource partitioning and coexistence between leopard and lion in Gir. Structural habitat heterogeneity (closed vs. Open) in Gir is a critical determinant of spatial partitioning between leopard and lion and hence crucial for coexistence of both the predators. Future change in habitat structure of Gir could have severe implications on coexistence of leopard and lion since both exist at very high densities. Since the scale of our study is limited to specific sites, there is an urgent need to assess how these two large predators coexist at a larger scale, such as home range.

References

1. Grassel SM, Rachlow JL, Williams CJ. Spatial interaction between sympatric carnivores: a asymmetric avoidance of an intraguild predator. Ecol Evol. 2015; 5:2762-2773.
2. Mills MGL. Conservation management of large carnivores in Africa. Koedeo. 1991; 34: 81-90.
3. Gause GF. 1934. The struggle for existence. Williams and Wilkins, Baltimore.
4. Wereszczuk A, Zalewski A. Spatial niche partitioning of sympatric stone martin and pine martin – avoidance of competition or selection of optimal habitat? PloS One 10(10): e0139852. https://doi.org/10.1371/journal.pone.0139852.
5. Schoener TW. Resource partitioning in ecological communities. Science 1974; 185:27–39.
6. Di Bitetti MS, De Angelo CD, Di Blanco YE, Paviolo A. Niche partitioning and species coexistence among Neotropical felid assemblage. Acta Ecol. 2010; 36: 403-412.
7. Ripple WJ, Estes JA, Beschta RL, Wilmers CC, Ritchie EG, Hebblewhite M, Berger J, Bodil E, Letnic M, Nelson MP, Schmitz OJ, Smith DW, Wallach AD, Wirsing AJ. Status and ecological effects of world's largest carnivores. Science. 2014;343:1241484.
8. Mac Nally RC. On assessing the significance of interspecific competition to guild structure. Ecology. 1983; 64:1646–1652.
9. Karanth KU, Srivathsa A, Vasudev D, Puri M, Parameshwaran R, Kumar NS. Spatio-temporal interactions facilitate large carnivore sympatry across a resource gradient. Proc Roy Soc B. 2017; 284:1-10.
10. Karanth KU, Sunquist ME. Prey selection by tiger, leopard and dhole in tropical forests. J Anim Ecol. 1995; 64:439-450.
11. Droge E, Creel S, Becker MS, Msoka J. Spatial and temporal avoidance of risk within a large carnivore. Ecol Evol. 2016; 7:1-11.
12. Mukherjee S, Zelcer M, Kotler B.P. Patch use in time and space for a mesopredator in a risky world. Oecologia. 2008; 159:661-668.
13. Bischof R, Ali H, Kabir M, Hameed S, Nawaz MA. Being the underdog: An elusive small carnivore with use space and time without enemies.: Space and time use by Altai mountain weasel. J Zoo. 2014; 293:40-48.
14. Swanson A, Caro T, Mostert HD, Mills MGL, Macdonald DW, Borner M, Masenga E, Packer C. Cheetahs and wild dogs show contrasting patterns of suppression by lions. J Anim Ecol. 2014; 83:1418-1427.
15. Sogbohossou E A, Kassa BD, Waltert M, Khorozyan I. Spatio-temporal niche partitioning between the African lion (Panthera leo leo) and spotted hyena (Crocuta crocuta) in western African savannas. Eur J Wild Res. 2018; 64: <https://doi.org/10.1007/s10344-017-1159-5>.
16. Pokheral CP, Wegge P. Coexisting large carnivores: spatial relationships of tigers and leopards and their prey in a prey-rich area in lowland Nepal. Ecoscience. 2019; 26:1-9.
17. Hayward MW, Slotow R. Temporal partitioning of activity in large African carnivores: test of multiple hypothesis. Afr J Wild Res. 2009; 39:109-126.
18. Vanak AT, Fortin D, Thaker M, Ogden M, Owen C, Greatwood S, Slotow R. Moving to stay in place: behavioural mechanism for coexistence of African large carnivores. Ecolology. 2013; 94(11):2619-2631.
19. Maputla NW, Maruping NT, Chimimba CT, Ferreira SM. Spatio-temporal separation between lions and leopards in the Kruger National Park and the Timbavati Private Nature Reserve, South Africa. Glob Ecol Cons. 2015; 3:693-706.
20. Ramesh T, Kalle R, Roselund H, Downs CT. Low leopard populations in protected areas of Maputaland: a consequence of poaching, habitat condition, abundance of prey, and a top predator. Ecol Evol. 2017; 7:1964-1973.
21. Mugerwa B, du Preez B, Tallents LA, Loveridge AJ, Macdonald DW. Increased foraging success or competitor avoidance? Diel activity of sympatric large carnivores. J Mamm. 2017; 98(5):1443-1452.
22. Balme G, Pitman RT, Robinson HS, Miller JRB, Funston PJ, Hunter LTB. Leopard distribution and abundance is unaffected by interference competition with lions. Behv Ecol. 2017; 28(5):1348-1358.
23. du Preez B, Purdon J, Trethowan P, Macdonald DW. Loveridge AJ. Dietary niche differentiation facilitates coexistence of two large carnivores. J Zoo. 2017; 302(3):149-156.
24. Miller JRB, Pitman RT, Mann GKH, Fuller AK, Balme G.A. Lions and leopards coexist without spatial, temporal or demographic effects of interspecific competition. J Anim Ecol. 2018; 87(6):1709-1726.
25. Zehra N, Khan JA, Chaudhary R. Food habits of large carnivores (Leopard and Lion) in Gir national park and sanctuary (GNPS), Gujarat, India. W J Zool. 2017; 12:67-81.
26. Khan JA, Chaudhary R, Zehra N. Monitoring structure, functioning and ecosystem services of a dry tropical forest ecosystem of Gir for conservation and management of ecosystem, prey population and predator. Annual Technical Report Submitted to MOEF&CC, New Delhi; 2019.
27. Jhala YV, Banerjee K, Chakrabarti S, Basu P, Singh K, Dave C, Gogoi K. Asiatic lion: Ecology, economics, and politics of conservation. Fron Ecol Evol. 2019; 7:1-21.
28. Swanson A, Arnold T, Kosmala M, Forester J, Packer. In the absence of a “landscape of fear”: How lions, hyenas, and cheetah coexist. Ecol Evol. 2016; 6:8534-8545.
29. Lovari S, Pokheral CP, Jnawali SR, Fusani L, Ferretti F. Coexistence of the tiger and the common leopard in a prey-rich area: the role of prey partitioning. J Zoo. 2015; 295:122-131.
30. Champion HG, Seth SK. A Revised Survey of the Forest Types of India, Government of India Press, Nasik; 1968.
31. Khan JA. Conservation and management of Gir Lion Sanctuary and National Park, Gujarat, India. Biol Cons. 1995; 73(3):183-188.
32. Ramesh T, Kalle R, Sankar K, Qureshi Q. Spatiotemporal partitioning among large carnivores in relation to major prey species in Western Ghats. J Zoo. 2012; 287:269-275.
33. Zar J. Biostatical Analysis. Pearson Publication House; 2006.
34. Ridout MS, Linkie M. Estimating overlap of daily activity patterns from camera trap data. [J Agr Biol Env Stat.](https://link.springer.com/journal/13253) 2009; 14(3):322-337.
35. Meredith M, Ridout M. overlap: Estimates of coefficient of overlapping for animal activity patterns. R package version 0.2.4. Available at: http://CRAN.R-project. org/package=overlap; 2014.
36. Qureshi Q, Shah N. Vegetation and habitat monitoring. In: Jhala YV (ed) Monitoring of Gir. A technical report submitted to the Gujarat forest department under GEF-India ecodevelopment program. Wildlife Institute of India, Dehra Dun, India; 2004.
37. Jacobs J. Quantitative measurement of food selection– a modification of the forage ratio and Ivlev’s electivity index. Oecologia. 1974; 14:413–417.
38. Stevens RD, Tello JS. Micro and macro habitat association in Mojave Desert rodent communities. J Mamm. 2009; 90:388-403.
39. Pianka ER. The structure of lizard communities. Ann Rev Ecol Evol Syst. 1973; 4:53–74.
40. Karanth KU, Sunquist ME. Behavioral correlates of predation by tiger (*Panthera tigris*) leopard (*Panthera pardus*) and dhole (*Cuon alpinus*) in Nagarahole, India. J Zoo. 2000; 250:255-265.
41. Rayan DM, Linkie M. Managing conservation flagship species in competition: Tiger, leopard and dhole in Malaysia. Biol Cons. 2016; 204:360-366.
42. Yang H, Zhao X, Han B, Wang T, Mou P, Ge J, Feng L. Spatiotemporal patterns of Amur leopards in northeast China: Influence of tigers, prey, and humans. Mamm Biol. 2018; 92:120-128.
43. MacArthur RW, Pianka ER. On optimal use of a patchy environment. Amer Nat. 1966; 100(916):603-609.
44. Bailey TN. The African leopard: a study of the ecology and behavior of a solitary felid. Columbia University Press, New York; 1993.
45. Khan JA. Ungulate habitat relationships in Gir forest ecosystem and its management implications. Ph.D. Thesis. Aligarh Muslim University, Aligarh, India; 1993.
46. Johnsingh AJT. Large mammalian prey-predators in Bandipur. J Bom Nat Hist Soc. 1983; 80:1 – 57.
47. Rafiq K, Jordan NR, Wilson AM, McNutt JW, Hayward MW, Meloro C, Wich SA, Golabek KA. Spatio-temporal factors impacting encounter occurrences between leopards and other large African predators. J Zool. 2019; <https://doi.org/10.1111/jzo.12746>.
48. Balme G, Hunter L, Slotow R. [Feeding habitat selection by hunting leopards *Panthera pardus* in a woodland savanna: prey catchability versus abundance](https://www.sciencedirect.com/science/article/pii/S0003347207001947). Anim Behv. 2007; 74 (3):589-598.
49. du Preez B, Hart T, Loveridge AJ, Macdonald DW. Impact of risk on animal behaviour and habitat transition probabilities. Anim Behv. 2015; 100:22–37.
50. Gorini L, Linnell JDC, Panzacchi M, Boitani L, Odden M, Nilsen E.B. Habitat heterogeneity and mammalian prey-predator interaction. Mamm Rev. 2011; 10.1111/j.1365-2907.2011.00189.x.
51. Jhala YV, Banerjee K, Basu P, Chakrabarti S, Gayen S, Gogoi K, Basu A. Ecology of lions in Gir PA and adjoining human dominated landscape of Saurashtra, Gujarat. Final Project Report (2011-2016) submitted to the Gujarat forest department. Technical Report, Wildlife Institute of India, Dehradun, India; 2016.

Table 1. Circular statistics of activity pattern of leopard, lion and their prey in Gir

| Variable | Leopard | Lion | Chital | Sambar | Nilgai | Wild Pig |
| --- | --- | --- | --- | --- | --- | --- |
| Number of observations | 289 | 153 | 631 | 101 | 33 | 50 |
| Mean vector | 23:11 | 01:20 | 11:30 | 15:17 | 11:04 | 13:52 |
| Circular variance | 0.62 | 0.535 | 0.489 | 0.787 | 0.445 | 0.714 |
| Standard error of mean | 00:41 | 00:21 | 00:27 | 01:14 | 00:26 | 00:55 |
| 95% confidence intervals | 21:49-00:32 | 00:39-02:02 | 10:36-12:23 | 12:50-17:43 | 10:11-11:56 | 12:04-15:41 |
| Rayleigh’s test | 14.08, p<0.001 | 51.6, p<0.05 | 30.34, p<0.05 | Z=4.59, p<0.05 | Z=30.44, p<0.05 | Z=30.34, p<0.05 |

Table. 2 Spatial and temporal overlap and association between leopard and lion and with their prey

| Temporal overlap and association Spatial overlap and association | | | | |
| --- | --- | --- | --- | --- |
|  | Overlap (95%CI) | Correlation coefficient (r) | Overlap | Correlation coefficient (r) |
| Leopard-lion | 80 (0.72-0.86) | 0.73* | 0.51 | .041 |
| Leopard-chital | 0.43 (0.34-0.44) | -0.69* | 0.40 | 0.34* |
| Leopard-Sambar | 0.69 (0.54-0.72) | 0.12 | 0.42 | 0.23* |
| Leopard-Nilgai | 0.37 (0.28-0.48) | -0.44* | 0.32 | 0.16 |
| Leopard-Wild pig | 0.53 (0.44-0.62) | -0.1 | 0.26 | 0.11 |
| Lion-chital | 0.36 (0.26-0) | -0.71* | 0.42 | .07 |
| Lion-Sambar | 0.61 (0.46-0.65) | -0.30 | 0.14 | -0.53* |
| Lion-Nilgai | 0.36 (0.26-0.47) | -0.63* | 0.26 | .09 |
| Lion-Wild pig | 0.44 (0.34-0.54) | -0.1 | 0.12 | -0.28* |

* p<0.05

Table 3 Percent habitat used by leopard, lion and their prey in different habitat

| Habitat | Leopard | Lion | Chital | Sambar | Nilgai | Wild pig |
| --- | --- | --- | --- | --- | --- | --- |
| MM | 30 | 35.3 | 15.4 | 26.1 | 5.1 | 11.5 |
| TAZ | 27.9 | 31.1 | 55.7 | 13 | 61.5 | 34.7 |
| M | 37 | 13.6 | 19 | 53.2 | 12.8 | 52.1 |
| T | 5 | 19.8 | 9.7 | 7.4 | 20.5 | 1.4 |
