# Supplementary material for: Spatio-temporal partitioning and coexistence between leopard (Panthera pardus fusca) and Asiatic lion (Panthera leo persica) in Gir protected area, Gujarat, India
Source: PLoS One. 2020 Mar 11;15(3):e0229045. doi: 10.1371/journal.pone.0229045 (PMC7065753; doi:10.1371/journal.pone.0229045)
Supplement: S4 Data — (DOCX) [file pone.0229045.s005.docx]

**Camera trap locations**

| **S.No** | **Camera ID** | **Lat** | **Long** |
| --- | --- | --- | --- |
| 1 | Hilly kehdi 5 | 70.5689 | 21.2350 |
| 2 | Nanava | 70.6066 | 21.1970 |
| 3 | Dedakadi to dudhala chella | 70.5836 | 21.2516 |
| 4 | Dadia ness | 70.6294 | 21.1969 |
| 5 | Valadara | 70.6456 | 21.1963 |
| 6 | Pilipat | 70.5949 | 21.1986 |
| 7 | Tapli grid | 70.5683 | 21.2042 |
| 8 | Juni alawani | 70.6405 | 21.2169 |
| 9 | Station Grid | 70.6285 | 21.2180 |
| 10 | Diificult grid | 70.6155 | 21.2215 |
| 11 | Kadeli | 70.5862 | 21.2133 |
| 12 | Raidi chella | 70.5766 | 21.2155 |
| 13 | Navi alawani | 70.6418 | 21.2342 |
| 14 | Dudhala ness | 70.5910 | 21.2350 |
| 15 | Kasia thana | 70.6469 | 21.2523 |
| 16 | Semardi ka chella | 70.6295 | 21.2566 |
| 17 | Route No 5 | 70.5688 | 21.2519 |
| 18 | Jambuthala | 70.6120 | 21.2514 |
| 19 | Khada_2 | 70.5538 | 21.2512 |
| 20 | Dargah Wali kehdi | 70.6261 | 21.2333 |
| 21 | Fishing cat kehdi | 70.6088 | 21.2369 |
| 22 | Bhambafod | 70.5823 | 21.1850 |
| 23 | Dedakadi to jambuthala road | 70.5966 | 21.2694 |
| 24 | Khada_1 | 70.5538 | 21.2369 |
| 25 | Dedakadi_1 | 70.5694 | 21.2694 |
| 26 | Alawani to barwaniya_2 | 70.6836 | 21.2172 |
| 27 | Alawani to barwaniya_1 | 70.6642 | 21.2209 |
| 28 | Barwaniya grid | 70.6888 | 21.2323 |
| 29 | Bheelagar water point | 70.7227 | 21.2341 |
| 30 | Gebiamba_1 | 70.6296 | 21.1626 |
| 31 | Gebiamba_2 | 70.6438 | 21.1653 |
| 32 | Ghum grid | 70.7506 | 21.2158 |
| 33 | Jhinjhuvania water point | 70.6611 | 21.1661 |
| 34 | Kamleshwar_1 | 70.6276 | 21.1774 |
| 35 | Kamleshwar thana | 70.6602 | 21.1960 |
| 36 | kankai_1 | 70.6873 | 21.1589 |
| 37 | Kankai-2 | 70.7054 | 21.1546 |
| 38 | Kankai_3 | 70.7246 | 21.1560 |
| 39 | Karamdadi | 70.7058 | 21.1793 |
| 40 | Karamdadi_rosadi_ness | 70.7185 | 21.1713 |
| 41 | Karamdadi check dam | 70.6884 | 21.2005 |
| 42 | Kasia trail | 70.6703 | 21.2311 |
| 43 | Kutiya | 70.7044 | 21.2151 |
| 44 | Kotiya to barwania | 70.7059 | 21.2321 |
| 45 | peeplawali aati | 70.6678 | 21.1830 |
| 46 | Ratanghuna | 70.6115 | 21.1843 |
| 47 | Semarwala board | 70.6479 | 21.1765 |
| 48 | Sitamadhi | 70.6865 | 21.1736 |
| 49 | Sukhi hiran 1 | 70.7044 | 21.1990 |
| 50 | Sukhi_hiran 2 | 70.7231 | 21.1967 |
